# Influence of technology adoption on farmers’ well-being: Systematic literature review and bibliometric analysis

**DOI:** 10.1016/j.heliyon.2024.e24316

**Published:** 2024-01-14

**Authors:** Mariani Abdul-Majid, Siti Aisyah Zahari, Norfaizah Othman, Suhaila Nadzri

**Affiliations:** a Faculty of Economics and Management, Universiti Kebangsaan Malaysia, Malaysia; b Faculty of Management and Muamalah, Selangor Islamic University, Malaysia

**Keywords:** Technology adoption, Farmer, Agriculture, Well-being, Systematic review

## Abstract

This study aimed at determining the effects of technology adoption on farmers' well-being. Specifically, we analysed and extended the current understanding of the topic by focusing on the concepts of technology adoption and well-being. The data were sourced from 30 papers that were selected based on specific criteria from the WoS and Scopus databases. They were acquired through a systematic search and quality appraisal process. The adopted technology can be grouped into sustainable agricultural practices and innovations, digital agriculture and information technology, precision farming and resource management, financial inclusion and agricultural transactions, remote sensing and satellite technology, and agricultural programs and initiatives. Most papers indicated that technology adoption improved farmers' well-being which was basically measured using productivity and income. The measure however lacked farmers' value judgments, such as happiness. Agricultural technology could have a mixed effect on farmers' well-being, depending on the type of technology adopted and the compatibility of farmers with technology in their agricultural practices. This study will assist researchers in developing an appropriate framework and strategy to improve farmers' welfare, which can alleviate poverty. These may further help policymakers identify the best technological innovations that can improve farmers' economic and social welfare, thereby facilitating the development of plans to meet farmers’ needs and interests. Finally, the study suggested future research directions for researchers and academics.

## 1 Introduction

The United Nations [1] had projected that the global population will surpass 9.7 billion people by 2050 and may even reach 11 billion by the end of the century. High population growth will increase the demand for food. Therefore, agriculture plays an important role in providing and maintaining an adequate food supply, thereby improving food security and nutrient availability for human consumption. To address these issues, numerous advancements and adjustments have been made in this field in previous years in terms of various farming methods, management practices, and technological innovations to help farmers increase agricultural productivity. Meanwhile, there is increasing recognition from organizations that technology can be the best solution to these challenges. Thus, the International Bank for Reconstruction and Development (IBRD) and the International Development Association (IDA) offer capital and support for the use of technology in the farming sector. The World Bank [2] reported that IBRD and IDA invested $6.7 billion in agriculture and related sectors. This investment was directed toward 157 projects aimed at improving the assets and services available to 9.7 million farmers, with the focus on helping 1.3 million farmers adopt more advanced agricultural technologies. In addition, educational programs have been launched by governments to encourage farmers to use advanced crop technology [3].

The advancement of digital technology has increased its use in farming and agricultural activities [4]. Artificial intelligence, smart farming, remote sensing, machine learning, device sensors, aerial images, moisture and temperature sensors, and GPS technology are some examples of technological innovations in the agricultural sector [5]. These technologies similarly have a significant impact on the agricultural business model, which can lead to a safer, more efficient, profitable, and environmentally friendly sector [6]. In addition, these can also enhance decision-making, thereby enabling improved risk and variability management to maximize yields and boost the entire economic system [7]. The World Bank [8] reported that crop yields have increased six-fold in the past 40 years following the adoption of technologies in the farming sector.

Beyond its economic impact, digital transformation in the agricultural sector will also benefit farmers in many ways, including enhancing their well-being [9]. In addition, the increasing usage of digital technologies and communication requires knowledge and skills to adapt to new norms. Human resource development is becoming an important concern when implementing IR 4.0 [10]. Low-skilled jobs will be taken over by autonomous machines in the future; therefore, workers must acquire new skills related to smart machine operation [11]. Skilled human resources are one of the most critical elements of the Industry Revolution (IR) 4.0 [12]. It is emerging as a very challenging issue in the palm oil industry. Stakeholders are reluctant to move toward the new technology due to the lack of understanding of its potential features and advantages, and they thus hesitate to invest in the new technology. Smallholders therefore faced financial hardship due to the increasing cost of living, exposure to poverty, social discrimination and inequality, and were subsequently unable to achieve economic well-being.

Until recently, a few criteria have been used to assess the effects of agricultural breakthroughs on farmers' well-being, including output [13], productivity [[14], [15], [16]], farm income [14,15], consumption expenditure [13], employment [17], and trade [18]. Moreover, technology adoption can contribute to sustainable farming systems [19]. Past studies have examined the impact of technology adoption on farmers’ well-being using various criteria and technological mechanisms [14,16,20]. However, the results vary depending on the concepts of well-being used and the analysis of technology adoption.

This study aimed at determining the effect of technology adoption on farmers' well-being. In this study, we systematically reviewed prior studies on this subject by conducting a systematic literature review (SLR) and bibliometric analysis. This process provides a comprehensive overview of the current state of knowledge, enabling policymakers, researchers, and practitioners to make informed decisions regarding the impact of technology adoption in agriculture specifically on farmers' well-being. An SLR helps elucidate the effects of technology adoption on farmers' well-being, offering a nuanced understanding of how different technologies affect different aspects of well-being. Our findings showed that technology can be categorized into sustainable agricultural practices and innovations, digital agriculture and information technology, precision farming and resource management, financial inclusion and agricultural transactions, remote sensing and satellite technology, and agricultural programs and initiatives. Although we identified mixed results, most studies however indicated that technology adoption improved farmers’ well-being. Most past studies referred to well-being as both productivity and income. Other studies have used different concepts of well-being, including per-capita consumption, environmental quality, and consumer/producer surplus. We further found that most studies were conducted in a single country while cross-country studies focused on developing countries. Opportunities for future research include comparative studies between developing and developed countries and the adoption of mixed quantitative and qualitative approaches.

This study contributes to the literature on agriculture economics and technology adoption by providing a comprehensive SLR on the effects of technology adoption on farmers' well-being. Vemireddy and Choudhary's [21] conducted a similar study, but focused only on female farmers. This study however will help researchers develop an appropriate framework and strategy to improve overall farmer welfare, which could alleviate poverty. It may further help policymakers identify the best technological innovations that will affect farmers' economic and social welfare, enabling them to develop plans that meet their needs and interests. Finally, this study provides future directions for researchers and academics.

The remainder of this study is organized as follows. Section 2 describes the methods used in this study. Section 3 presents the findings of this study. Section 4 summarizes the discussion and offers suggestions for future research, followed by Section 5, which presents the measurement of well-being. Section 6 summarizes the main conclusions of the study, including the characteristics of the chosen study and the impact of technology adoption on farmers’ well-being.

## 2 Methodology

To create a good SLR, our study used the preferred reporting items for systematic reviews and meta-analyses (PRISMA) approach. The PRISMA model is a well-known standard for publishing SLR papers [22,23]. It aids writers in explicitly defining research topics for the systematic review. Additionally, the PRISMA model assists writers in determining the inclusion and exclusion criteria to ensure that the chosen article is pertinent to the study's context. The PICo model served as the foundation for developing an appropriate research question for this study. There are three main ideas in PICo; population or problem, interest, and context. Based on these ideas, the authors have included all the three concepts in the review; mainly farmers' (Population) influence of technology adoption (Interest), and farmers' well-being (Context). These concepts help the authors to express the main research question, “*What are the influences of technology adoption on farmers' well-being*?"

Next, we applied the following research questions (RQ) to achieve the objective of this study:

RQ1 What are the characteristics of these studies? Specifically, what are the research publication trends in this area, the most productive journals, the most contributing countries, country collaboration networks, and their affiliations?

RQ2 What are the theories employed in studying the influence of technology adoption on farmers' well-being?

RQ3 What are the influences of technology adoption on farmers' well-being?

RQ4 What are the measurements of well-being?

**Fig. 1:**
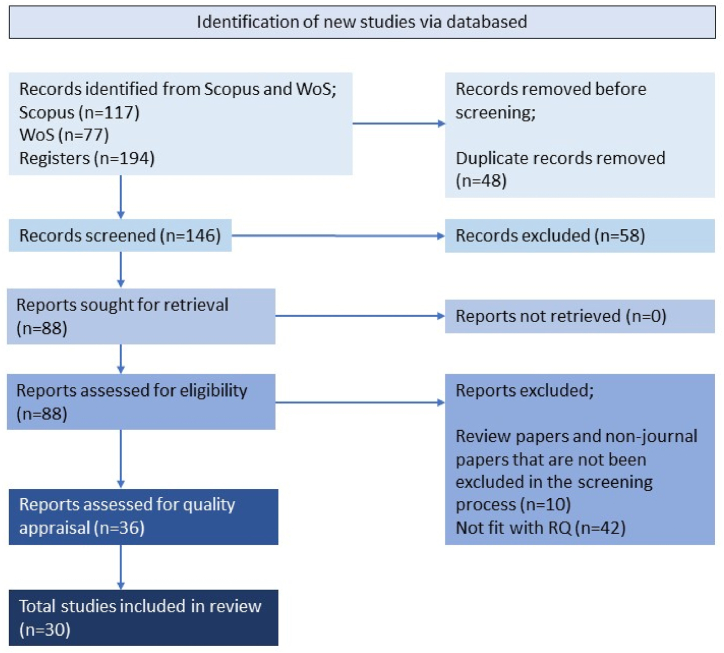
Article extraction and searching process.

*Sources*: Authors' compilation based on PRISMA model (2023)

Fig. 1 explains the extraction of selected papers and the search process. This study began with an identification process in which the authors identified relevant documents from the database. We selected Scopus and the Web of Science (WoS) as the main databases for this study since they are globally the leading citation databases. In this process, we used keywords related to technology, farmers, and well-being and their synonyms to build a search string. The search functions integrated into the primary database were used to assess the combinations of these relevant keywords. To expand the scope of the database search, we used field code functions, phrase searching, wildcards, truncation, and Boolean operators. In total, 194 papers were retrieved from the identification process. The final search string is as follows: (("technolog*" OR “machiner*" OR “robot*" OR “automation" OR “artificial intelligence" OR “4IR" OR “high tech" OR “Mechanization" OR “sensor*" OR “agriculture 4.0″ OR “agritech" OR “drone*" OR “m-money") AND ("Farmer*" OR “smallholder*" OR “Rancher*" OR “agricultural worker*" OR “cultivator*" OR “Harvester*" OR “cropper*" OR “breeder*" OR “tiller*") AND ("economic well-being" OR “economic well being" OR “financial well-being" OR “financial well-being" OR “economic welfare" OR “financial welfare" OR “economic prosperity" OR “financial prosperity" OR “welfare economic" OR “social well-being" OR “social well-being" OR “social prosperity" OR “social welfare" OR “well-being of society" OR “public welfare" OR “public well-being")).

The second step was screening. Throughout the screening process, we included and excluded papers based on specific inclusion and exclusion criteria. The first criterion was document types, whereby only papers published in a journal were screened to ensure the quality of the study. In addition, we only included papers published in English to explicitly understand the study. We identified 136 papers using this selection strategy and excluded 48 duplicated papers. Thus, 88 papers were considered eligible for the next step.

During the eligibility process, we reviewed the title and abstract of each article to ensure that it was suitable for the current study. Consequently, 52 papers were excluded since they were not in line with the research objective. Thus, 36 papers were considered for review.

The remaining papers were evaluated, reviewed, and analysed through a quality appraisal process handled by two experts. Twenty-five papers were categorized as high quality, five as moderate quality, four as low quality, and two were rejected because they were review papers. Only papers categorized as high or moderate were included in the subsequent eligibility process. Thus, 30 papers were eligible for data extraction. Through this process, we extensively analysed the abstract, methodology, findings, discussion, and conclusion and identified the theme and subthemes. These papers are listed in the reference list with their corresponding codes ranging from 1 to 30.

## 3 Findings

In answering RQ1, we elaborated on the research publication trend in this area, the most productive journal, most contributing countries, country collaboration network, and affiliation in this area.

### 3.1 Characteristics of the study

Table 1 classifies the papers according to their year of publication. In terms of research publication trend, these papers began publication in 1970. The subject gained attention from researchers worldwide since 2017. The year 2020 and 2021 showed the highest number of publications in this area, covering approximately 66.6 % of the total papers. Therefore, the current study is included in a recent research topic.

**Table 1:** Publication years.

Publication years	Number of publications	Percentage of total publications (%)
2022*	2	6.7
2021	6	20.0
2020	6	20.0
2019	1	3.3
2018	1	3.3
2017	4	13.3
2016	1	3.3
2015	1	3.3
2014	2	6.7
2005	2	6.7
1998	1	3.3
1995	1	3.3
1991	1	3.3
1970	1	3.3
Total	30	100

Notes: *As of August 2022.

We also categorized the selected papers on the basis of their published journals. A total of 34 well-known journals were included in this study. All selected papers were listed in either the Journal Citation Reports (JCR) or Scimago Journal Rank (SJR) or both. Some journals were ranked as NA because they were not assigned to any quartile in the previous year. Most papers were ranked in quartile 1 (Q1), whether in the JCR or SJR indices. Nineteen papers were listed in the Q1 SJR index, whereas 13 papers were listed in the Q1 JCR index. This showed that the selected papers for the review process were published in high-ranked and prestigious journals. Table 2 classifies the papers based on their published journals. The most productive journals are the Journal of Rural Studies and PloS ONE.

**Table 2:** Number of studies classified by published journal and quartile.

Source titles	Record count	% of 30 papers	2021 SJR index	2021 JCR index
1. Journal of Rural Studies	2	6.7	1.29 (Q1)	5.16 (Q1)
2. PLoS ONE	2	6.7	0.85 (Q1)	3.75 (Q2)
3. International Journal of Environmental Research and Public Health	1	3.3	0.81 (Q1)	4.61 (Q2)
4. Africa Journal of Management	1	3.3	0.42 (Q2)	4.94 (Q4)
5. American Economic Journal: Applied Economics	1	3.3	10.08 (Q1)	7.96 (Q1)
6. Remote Sensing	1	3.3	1.28 (Q1)	5.35 (Q2)
7. Food Policy	1	3.3	1.93 (Q1)	6.08 (Q1)
8. Agriculture and Human Values	1	3.3	1.01 (Q1)	4.91 (Q1)
9. Library Philosophy and Practice	1	3.3	NA	NA
10. Journal of Integrative Agriculture	1	3.3	0.75 (Q1)	4.38 (Q1)
11. Environmental Economics and Policy Studies	1	3.3	0.60 (Q2)	NA
12. Manufacturing and Service Operations Management	1	3.3	7.13 (Q1)	7.01 (Q1)
13. Journal of Environmental Economics and Policy	1	3.3	0.55 (Q2)	0.42 (Q4)
14. American Journal of Agricultural Economics	1	3.3	1.86 (Q1)	3.76 (Q2)
15. Technology in Society	1	3.3	1.14 (Q1)	6.89 (Q1)
16. International Journal of Scientific and Technology Research	1	3.3	NA	NA
17. Journal of the Association of Environmental and Resource Economists	1	3.3	2.80 (Q1)	3.92 (Q1)
18. Heliyon	1	3.3	0.55 (Q1)	3.78 (Q2)
19. World Trade Review	1	3.3	0.86 (Q1)	1.71 (Q3)
20. Sustainability (Switzerland)	1	3.3	0.66 (Q1)	3.89 (Q2)
21. International Journal of Cognitive Informatics and Natural Intelligence	1	3.3	0.24 (Q4)	NA
22. World Development	1	3.3	2.30 (Q1)	6.69 (Q1)
23. Agricultural and Food Economics	1	3.3	0.85 (Q1)	2.87 (Q1)
24. Journal of Environmental Management	1	3.3	1.48 (Q1)	8.91 (Q1)
25. Journal of Development Studies	1	3.3	0.95 (Q1)	2.52 (Q2)
26. Journal of Cleaner Production	1	3.3	1.92 (Q1)	11.07(Q1)
27. Journal of Central European Agriculture	1	3.3	0.20 (Q4)	0.23 (Q4)
28. Climate Risk Management	1	3.3	1.29 (Q1)	5.27 (Q1)

Note: NA refers to not available.

In Table 3, the studies are classified on the basis of their country samples. Most of the studies were conducted on the basis of a single-country sample, which covered approximately 81 % of the total papers. The highest number of publications were conducted in the United States, Nigeria, and China (3/36), followed by Indonesia (2/36) and Zambia (2/36). India, Rwanda, Mozambique, Denmark, Ethiopia, the Czech Republic, Australia, Sri Lanka, Bangladesh, Mexico, Kenya, and the Philippines recorded one paper each. Only 13.9 % (5/36) of the total studies were conducted using multi-country samples comprising Ghana, Senegal, and Mali; the United States and European Union; Sub-Saharan Africa, Argentina, Bolivia, Peru, Brazil, China, North Korea, Paraguay, and Africa; and Canada and the United States.

**Table 3:** Number of papers by country.

Country	Papers	No. of papers
Single-country sample		
United States	15, 29, 30	5
Nigeria	2, 18, 27	3
China	3, 4, 9	3
Indonesia	12	1
Zambia	13, 25	2
India	14, 16	2
Rwanda	6	1
Mozambique	7	1
Denmark	5	1
Ethiopia	8	1
Czech Republic	10	1
Australia	11	1
Sri Lanka	19	1
Bangladesh	20	1
Mexico	22	1
Kenya	24	1
The Philippines	28	1
Multi-country sample		
Ghana, Senegal, and Mali	1	1
United States and European Union	26	1
Sub-Saharan Africa	21	1
Argentina, Bolivia, Peru, Brazil, China, North Korea, Paraguay, and Africa	17	1
Canada and the United States	23	1

Note: This table lists the number of publications per country/region. The paper numbers (1–30) correspond to the list provided in the Appendix.

**Fig. 2:**
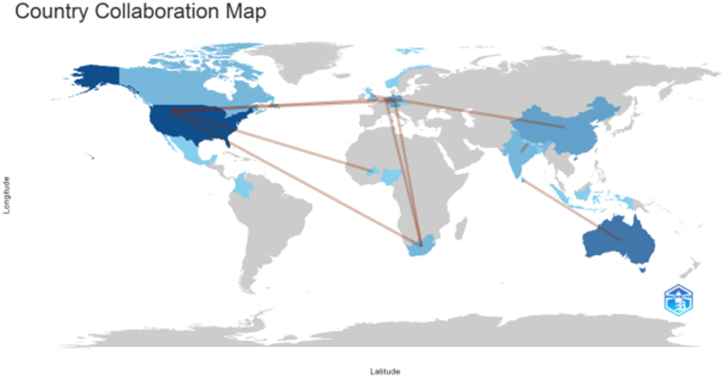
Country collaboration network.

*Sources*: Authors' work using R-programme (2023)

Fig. 2 illustrates the global maps on country collaboration produced using the R program. The frequency of encounters between nations serves as a proxy for cooperation. For instance, authors from China and the United Kingdom have worked together once, as have authors from Australia and Sri Lanka. The same applies for authors from the Netherlands and South Africa, India and Nepal, South Africa and the Netherlands, the United States and Burkina Faso, the United States and Germany, the United States and the Netherlands, and the United States and South Africa. These findings demonstrate cross-national cooperation, which is helpful for understanding international research issues and commercial and diplomatic ties.

**Fig. 3:**
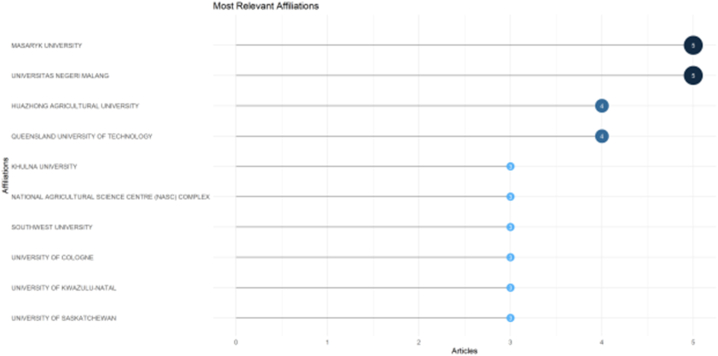
Most relevant affiliation.

*Sources*: Authors' work using R-programme (2023).

Fig. 3 shows the authors' affiliations. Authors from Masaryk University and Universitas Negeri Malang have published five papers. Huazhong Agricultural University and Queensland University of Technology have published four papers. They may be renowned centres of knowledge and competence in their respective domains, and these linkages suggest a considerable contribution to research and academic output. Such affiliations frequently serve as markers of institutions that are actively involved in intellectual and research endeavours, making them valuable centres for information sharing and academic cooperation related to technology and farmers’ well-being.

### 3.2 Theories

To answer RQ2, we have summarized in Table 4 the theories employed in relation to the influence of technology adoption on farmers’ well-being. These can be broadly grouped into economic theories, farming and agricultural models, and other agricultural and development models. These theories provide the primary tools for understanding and analysing economic dynamics. Domestication theory examines technology in the complex settings of everyday life, focusing on interpersonal relationships, social background, changes, and continuities [24]. There are four phases of domestication perspective, including appropriation, objectification, incorporation, and conversion [24], in adopting technology in daily activities. The resource-based view theory examines how businesses use their resources to obtain an advantage [25]. Three papers do not specifically mention the economic theories employed. A specialized economic model that handles irreversible advantages is also introduced by the Santaniello Theorem of the Irreversible Advantages Model. The theorem explicitly considers changes in producer and consumer surplus, which are common welfare indicators [26]. This model is particularly pertinent in situations where economic decisions are being made. Collectively, these theories advance the study of economics by illuminating economic trends, decisions, and strategies across a range of fields.

**Table 4:** Theoretical background.

Theoretical background	Papers	No. of papers
Economic theories		
Domestication theory	6	1
Resource-Based View theory	17	1
Economic theory	7, 8, 29	3
Santaniello Theorem of the Irreversible Benefits Model	21	1
GTAP Model (based on neoclassical economic theory)	26	1
Growth-machine theory	15	1
Farming and Agricultural Models		
APSIM Model	28	1
SCUAF Model	28	1
Non-separable model	25	1
Random utility and demand theory	21	1
Cobb –Douglas model	20	1
Other Agricultural and Farming Development Model		
Theory of change	1	1

The second group, farming and agricultural models, includes the SCUAF model-separable model, random utility and demand theory, and the Cobb–Douglas model. To simulate, analyse, and anticipate many elements of farming and agricultural systems, agricultural models provide organized frameworks essential to the study of agriculture. The APSIM and SCUAF models simulate crop growth, pest management, and farm management [27]. In contrast to the random utility and demand theory, which examines consumer preferences and decisions regarding agricultural products, non-separable models analyse the interdependencies between various farming factors. A well-known production function model, the Cobb–Douglas model, aids economists and decision-makers in comprehending the variables affecting agricultural production.

Finally, another agricultural and farming development model is the theory of change, which includes four steps that outline the desired outcomes or changes in farming practices; namely inputs, immediate outcomes, intermediate outcomes, and final outcomes [14]. It offers a guide for determining how different activities and policies may affect rural populations and ecosystems.

### 3.3 Influence of technology adoption on farmers’ well-being

In answering RQ3, Table 5 analyses the technology mechanism and Table 6 evaluates the influence of technology adoption on farmers’ well-being**.** The tables are divided into six main panels based on the technology mechanisms used by the farmers. Panels A, B, C, D, E, and F indicate sustainable agricultural practices and innovations, digital agriculture and information technology, precision farming and resource management, financial inclusion and agricultural transactions, remote sensing and satellite technology, and agricultural programs and initiatives**,** respectively.

**Table 5:** Technology mechanism classified by the number of papers.

Technology mechanism	Papers	No. of papers
Panel A: Sustainable Agricultural Practices and Innovations	1, 2, 3, 7, 8, 9, 13, 16, 17, 20, 21, 23, 25, 26, 28, 29	16
Green revolution technology	7	1
Conservative agricultural technology	9, 17	2
Zero tillage	16	1
IPM technology	20	1
GE crop	21	1
GM technology	23, 26	2
Hybrid seed	25	1
Improved variety of seeds	1	1
IMV	2	1
Crop diversification	2	1
CSAPs	3	1
Stall feeding	8	1
Agriculture intensification	13	1
Hedgerow intercropping	28	1
Ridge till	29	1
Panel B: Digital Agriculture and Information Technology	11, 12, 14, 18	4
Digital farming	12	1
Digital technology	11	1
Decision trees and SVM	14	1
ICT	18	1
Panel C: Precision Farming and Resource Management	15, 19, 22, 27	4
Shale development	15	1
IIAD	19	1
Biofertilizer	22	1
Cassava and maize technology	27	1
Panel D: Financial Inclusion and Agricultural Transactions	6, 24	2
Mobile money	6, 24	2
Panel E: Remote Sensing and Satellite Technology	10	1
Landsat 8	10	1
Sentinel 2a/b	10	1
Panel F: Agricultural Programs and Initiatives	1, 5	2
WAAAP technology	1	1
Agricultural innovation	5	1

Note: This table lists the number of publications per country/region. The paper numbers (1–30) correspond to the list provided in the Appendix.

**Table 6:** Technology mechanisms and their effects on farmers’ well-being.

Technology mechanism	Effects	Papers	No. of papers
Panel A: Sustainable Agricultural Practices and Innovations			
Green revolution technology	+	7	1
Conservative agricultural technology	+	9	1
	NA	17	1
Zero tillage	NS	16	1
IPM technology	+	20	1
	–	20	1
GE crop	+	21	1
	–	21	1
GM technology	+	23, 26	2
	–	23	1
Hybrid seed	+	25	1
	–	25	1
Improved variety of seeds	+	1	1
IMV	+	2	1
Crop diversification	+	2	1
CSAPs	+	3	1
Stall feeding	+	8	1
	–	8	1
Agriculture intensification	+	13	1
Hedgerow intercropping	+	28	1
	–	28	1
Ridge till	+	29	1
	–	29	1
Panel B: Digital Agriculture and Information Technology			
Digital farming	+	12	1
Digital technology	NS	14	1
Decision trees and SVM	+	11	1
ICT	+	18	1
Panel C: Precision Farming and Resource Management			
Shale development	+	15	1
IIAD	+	19	1
Biofertilizer	+	22	1
Cassava and maize technology	+	27	1
Panel D: Financial Inclusion and Agricultural Transactions			
Mobile money	+	6, 24	2
	–	24	1
Panel E: Remote Sensing and Satellite Technology			
Landsat 8	NS	10	1
Sentinel 2a/b	NS	10	1
Panel F: Agricultural Programs and Initiatives			
WAAPP technology	+	1	1
	–	1	1
Agricultural innovation	+	5	1
	–	5	1

Note: NS refers to Not Significant, NA refers to Not Available. This table lists the number of publications per country/region. The paper numbers (1–30) correspond to the list provided in the Appendix.

Table 6 categorizes the analysis on the basis of the effect and relationship of technology mechanisms on farmers’ well-being. The following subsection discusses related studies.

#### 3.3.1 Sustainable agricultural practices and innovation

The first theme developed from related studies was sustainable agricultural practices and innovations, which further included 15 sub-themes; green revolution technology, conservative agricultural technology, zero tillage, integrated pest management (IPM) technology, genetically engineered (GE) crop, genetically modified (GM) technology, hybrid seed, improved variety seed, improved maize-variety (IMV), crop diversification (CD), climate-smart agricultural practices (CSAPs), stall feeding (SF), agriculture intensification, hedgerow intercropping, and ridge till. Carter et al. [13] studied the effect of green revolution technology adoption on living standards. Measured by per-capita daily consumption, green revolution technology has shown a positive impact. This indicated an indirect positive relationship with agriculture, which improved agricultural profit and living standards of farmers in Mozambique.

Conservation agricultural technology refers to deep tilling, no-till farming, shrimp and fish culture, returning straw to the field, and less tillage on farms [28]. Yang and Sang [29] measured the impact of conservation agricultural technology on farmers' well-being through income, social development, and ecological environment improvement. There was a significant positive correlation between conservation agricultural technology and income and social development. However, ecological environmental improvement showed a negative relationship. Dyck and Silvestre [25] studied a multi-country sample, including Argentina, Bolivia, Peru, Brazil, China, North Korea, Paraguay, and Africa. The study measured the relationship between conservation agricultural technology and financial and socio-ecological well-being with the objective of increasing farmers’ well-being.

Zero tillage is a good sustainable intensification technology for farmers in Bihar, India [30]. Adopting zero tillage increases wheat yield and consequent profitability, and lowers greenhouse gas emissions from agricultural equipment [30]. However, Afrin et al. [31] showed a positive and negative relationship between IPM technology and well-being. A positive correlation was found between IPM technology and health and environmental quality. A possible explanation for this might be that safer pesticides have a lower harmful impact on biodiversity deterioration and soil and groundwater contamination [31]. However, revenue decreased by following adoption of the technology.

GE crops were discussed by Wesseler et al. [26] for sub-Saharan Africa, namely, disease-resistant cooking banana (matoke), insect-resistant cowpea, and corn (maize). Further analysis showed that GE crops reduced producer and consumer surpluses and had a substantial impact on malnutrition. Two studies measured the impact of GM technology on farmers’ well-being. First, Smyth et al. [32] measured GM technology in relation to consumer surplus, producer surplus, productivity, and cost per unit of output. The results showed that GM technology could contribute to a reduction in producer surplus. Simultaneously, it reduced productivity because a smaller area could be planted with GM crops and costs per unit of output increased. In the second study, Jackson and Anderson [33] measured the effect of GM technology on total factor productivity, income, price, and poverty alleviation in a multi-country sample. The analysis revealed that GM technology correlated well with total factor productivity, income, and poverty alleviation. Nevertheless, the price declined after the European Union imposed a moratorium on imports from countries that adopted GM. Generally however, it can be suggested that GM technology has improved the overall welfare of farmers.

Crossing two genetically different plants creates a hybrid seed [34]. The adoption of hybrid seeds is related to low poverty and a greater asset base and wealth in terms of livestock and machinery [35]. Hybrid seeds increase the total household income, the value of all productive assets, the value of farms and processing equipment, and the total number of livestock units owned by farmers [35]. Thus, the severity of poverty and relative deprivation regarding overall assets are negatively correlated with hybrid seeds.

The improved seed variety in maize is complementary to WAAPP and other technologies. Farmers started using improved varieties of seeds after joining WAAPP. Improved seed is one of the main aims of WAAAP activities for farmers to enhance total production, yield productivity, and sales [14]. Moreover, IMVs and CDs have shown a significant positive impact on well-being in terms of productivity and net farm income [15]. Those that used IMVs alone and IMVs plus the CD packages showed the largest production gains, respectively [15]. Farmers who adopted IMVs and CD packages gained more income than those who did not. The enhancement in their net income was due to the higher yield productivity in maize production.

CSAPs include improved seedling varieties, improved irrigation facilities, adjusted production timing, adopted zero tillage, applied soil testing, developed fertilizer, and plant green manure [36]. Liang et al. [36] reported a significant positive correlation between CSAPs, rice yield, and farmers’ net income. Farmers who implemented CSAPs withstood climate change, thus enhancing their productivity and net income.

SF additionally showed mixed results on the well-being of farmers. SF had a positive effect on milk production and productivity, lactation period, consumption, manure use, likelihood of participating in animal sale markets, number of plants, and propensity to grow trees. However, the findings revealed that SF reduced its hiring of workers and purchased animal feed [16]. Although it decreased the overall number of animals in the herd, it increased the likelihood of keeping a milking cow. Agricultural intensification is the sixth subtheme of agricultural practice. Stacy [37] described agricultural intensification as a process of change in farm management methods and crop and non-crop vegetation. Goeb et al. [38] measured agricultural intensification with the use of pesticides. It has shown a negative effect on social welfare relating to farmers’ health and environmental externalities due to their imperfect knowledge of the risks posed by agricultural inputs.

Next, we refer to Nelson and Cramb [27], who discussed hedgerow intercropping in the Philippines, which produced a mixed effect on net income and thereby farmers' well-being. As there was less soil erosion and maize yields were maintained over the long term, the net returns from hedgerow intercropping were high. However, because of establishment expenses, hedgerow intercropping had shorter net returns than continuous and fallow open-field farming. Lastly, Lighthall and Roberts [39] measured the relationship between technology and farmers’ well-being using ridge tilling. A ridge till refers to a new tillage system for low-chemical input systems of corn or soybean production [39]. In this study, welfare was measured through profit, exposure to pesticides, and production risk. Ridge till improves profit and production risk. However, it has a negative impact on farmers because of exposure to pesticides. The positive effect is due to the reduction in chemical hazards and soil erosion and reduction in the time required for production and cultivation. Thus, this theme illustrates that farmers tend to integrate agricultural practices with modern technology to improve their overall well-being.

#### 3.3.2 Digital agriculture and information technology

Digital agriculture and information technology are the second theme identified in this study. The theme includes digital farming, digital technology, decision trees, support vector machines (SVMs), and information and communication technology (ICT). Digital farming, such as precision farming, can contribute to climate control and nutrition. Furthermore, investment in digital farming can create job opportunities for less capable segments, which can enhance the social welfare of farmers [40]. It indirectly has a positive impact on agricultural productivity. The agriculture sector contributes the most to the total gross regional domestic product in the Pangalengan sub-district of Bandung, Indonesia. Moreover, digital technologies support trade in agriculture and food products [18]. It could be in the form of a blockchain that could connect buyers and farmers directly [62]. Sowmya et al. [41] discussed the importance of using decision trees and SVMs. These modern tools measured rainfall, humidity, and temperature to help farmers in India choose the correct crop. This led to higher crop growth and well-being of the society.

The last subtheme in Digital Agriculture and Information Technology is ICT. According to Tijjani et al. [42], ICT performs several key functions in empowering women, including providing business opportunities, breaking down social isolation, linking women to inputs and markets, aiding small and medium enterprises, reducing poverty and illiteracy, and increasing the income and savings. ICT has improved the income and savings of rural women. It has a positive effect on income and savings because it offers entrepreneurial opportunities for rural women. ICT was proven to break the isolation of rural women in Nigeria by enhancing their knowledge regarding agriculture, thus leading to better decision-making and the ability to participate in development programs such as those for building skills and assisting small and medium enterprises. ICT has improved the well-being of Nigerian society by eradicating poverty, removing social and economic isolation, improving decision-making, and enhancing involvement in program design [42]. Nonetheless, the level of education of farmers could greatly influence the absorption of technology in the agricultural sector [43]. Hence, digital agriculture and information technology provide positive results on farmers’ well-being.

#### 3.3.3 Precision farming and resource management

The third subtheme is precision farming and resource management. The theme further includes shale development, irrigation-induced agricultural development (IIAD), bio fertilizer, cassava and maize technology, and mechanical tomato harvesting. Shale development has a range of consequences for farmers' well-being. Small et al. [44] defined shale development as the process of obtaining oil and natural gas from a shale formation. The process, known as hydraulic fracturing or ‘fracking’, entails injecting a solution of water, sand, and chemicals into a shale formation at high pressure to generate fractures that release the trapped hydrocarbons. Owing to the possible environmental impact of shale development, it has recently generated considerable discussion and controversy. Crowe [45] reported that shale development has a negative impact on the environment because it results in poor water quality that endangers cattle and could potentially reduce farmers' revenue. Technology that negatively impacts the environment, resulting in farmers' lower income, can also be found in the literature [61]. However, shale development also brings about positive outcomes in terms of farmers' financial well-being. The income provided through lease and royalty payments from shale development can generate long-term beneficial effects on agriculture and rural areas.

Additionally, Athukorala and Wilson [46] found a relationship between IIAD and welfare in terms of changes in consumer surplus, producer surplus, and total surplus. Investment in the IIAD has a positive impact on the overall welfare of farmers. In another study, Barragán-Ocaña and del-Valle-Rivera [47] indicated that bio-fertilizer technology could boost land productivity, reduce environmental impacts, and promote social development. The positive relationship is probably related to the fact that biofertilizers are both affordable and environmentally friendly and that their regular use improves soil fertility [48].

In Nigeria, Omobolanle [49] analysed the net benefits of cassava and maize technology in terms of income and productivity. Strong evidence of a positive relationship between these technologies and well-being was found when sustained users earned higher income and, consequently, a better standard of living [49]. Lastly, Schmitz and Seckler [17] established that gross social return (GSR) for tomatoes increased after the adoption of the mechanical tomato harvester. However, the societal costs associated with breakthroughs such as the tomato harvester would be significantly reduced [17] since the machine reduces the work required to manually harvest the tomato.

#### 3.3.4 Financial inclusion and agricultural transactions

The fourth theme is financial inclusion and agricultural transactions which concerns mobile money. This refers to monetary transactions that can be accessed online through m-money applications on various platforms [24]. Uwamariya et al. [24] and Kikulwe et al. [46] maintained that mobile money is a good financial inclusion and agricultural transaction tool for farmers. Uwamariya et al. [24] reported that m-money enabled farmers in rural Rwanda to digitally save money to meet future expenses, leading to an enhancement in their social welfare. Kikulwe et al. [50] stated that the use of m-money by farmers in Kenya was associated with significantly higher per-capita income and household income. Simultaneously, it produced a significant impact on remittances received, spending, and total banana production, resulting in higher profits. Farmers who adopted m-money gained 35 % over non-users.

#### 3.3.5 Remote sensing and satellite technology

The fifth subtheme is Remote Sensing and Satellite Technology, which is related to Landsat 8 and Sentinel 2a/b. The two data facilities had a significant impact on yield productivity, which increased economic prosperity [51]. They are also used to measure yield productivity in order to minimize the pollution caused by the fertilizers used by farmers [51,52].

#### 3.3.6 Agricultural programs and initiatives

The last theme developed from this study was agricultural programs and initiatives, which further comprised two sub-themes: WAAAP technology (the West Africa agricultural productivity program) and agricultural innovation. The main goal of these technologies is to ease supply-side constraints by exposing farmers to new technology [14,53]. The innovations disseminated by WAAPP and agricultural technology from other extension programs may complement one another [14]. Technology had a significant and intermediate effect on household agricultural production. WAAPP technology, seed, and agricultural technology from other extension programs enhance the income level of farmers. Brahmi et al. [63] found that some innovations are consistently integrated into the financial inclusion domain. However, the effect of technology on consumption and food security was not statistically significant due to the limited impact of technology deployment [14].

Agricultural innovation enhances social welfare. This was supported by Akkaya et al. [54] who studied Danish government support for agricultural innovation. Subsidies and taxes are examples of government intervention that have been examined for their effects on the adoption of new production techniques, producer profitability, consumer surplus, and return on government investment. The authors suggest that government intervention involving subsidies and taxes helped farmers utilize agricultural innovation in their practices, thereby enhancing their social welfare.

To summarize, technology could have either a positive or negative impact on farmers’ well-being, depending on the type of technology adopted and the compatibility of farmers with technology in their agricultural practices.

### 3.4 Well-being measurement

To answer RQ4, this section discusses various measurements of farmers’ well-being used in the studies included in this systematic review. We classified well-being into four main dimensions; general well-being, economic well-being, social well-being, and environmental well-being (refer Table 7).

**Table 7:** Measurements of well-being in existing studies.

Well-being indicator	Papers	No. of papers
General well-being	6, 13, 14, 15, 17, 19	6
Economic well-being		
Income	1, 2, 3, 4, 9, 18, 24, 25, 26, 27	10
Consumption	1, 7, 8, 19	4
Profitability	5, 16, 19, 20, 21, 23, 24, 27, 28, 29	10
Productivity	1, 2, 8, 10, 12, 22, 23, 24, 26, 27, 28	11
Poverty	8, 18, 25, 26	4
Saving and Investment	1, 5, 18, 24, 30	5
Employment and Entrepreneurship	1, 8, 18	3
Market Linkage	1, 3,5, 8, 18, 29	6
Social well-being		
Health and Nutrition	12, 13, 20, 21	4
Education and Skills	18	1
Social Development	9, 18, 22, 30	4
Environmental well-being		
Environmental Quality	9, 13, 15, 20, 22	5
Agricultural Practices	8, 20, 24, 29	4
Sustainable Practices	8, 25	2

General well-being is the most commonly used term that may refer to economic, financial, or social well-being. Economic well-being is divided into eight subthemes. Productivity, profitability, and income are the most frequently used metrics to evaluate economic well-being. Most studies (11/30) used productivity to measure well-being (row 1, column 2). Labour productivity is the simplest and most frequent way to define productivity, which is measured as homogenous physical output per unit of labour input [57]. Income is the second most widely used proxy for measuring well-being. High income may represent higher well-being.

The third most common measure of economic well-being is profitability which, in this study, refers to consumer surplus [26,32,46,54], producer surplus [26,32,46], profit [30,39,50,54], and return on government expenditure [5]. Consumer surplus is a social welfare indicator used to measure consumer benefits. In other contexts, money generated through trade or business after covering the costs of producing and delivering goods and services is referred to as profit [58]. In contrast, producer surplus refers to the difference between the price a producer receives for specific goods or services and the minimum price they would have been willing to accept. Specifically, it is the amount of money a producer earns over and above what they need to cover their production costs. The consumer surplus is an indicator of poverty alleviation aimed at permanently raising the population out of poverty.

Market linkages, saving and investment, consumption, poverty and employment, and entrepreneurship are part of the economic well-being measures adopted in past studies. Consumption expenditure is a good indicator of well-being because it indicates how a person spends their income in daily life. Similarly, savings are also a good indicator that measures the remaining money after subtracting a person's spending and consumption expenditures. Bambio et al. [14] measured well-being through sales which are part of employment and entrepreneurship and categorized as intermediate outcomes.

Social well-being was categorized into health and nutrition, education and skills, and social development. Two studies [31,38] employed health levels as measure of well-being. Freeman [59] asserted that the best way to calculate the health cost of pollution was to use a health production function. Pollution leads to morbidity costs, which have an impact on individual utility resulting in discomfort and anguish, illness, lost workdays, and medical expenses. Two studies [26,60] further used nutrition to measure farmers' well-being. Wesseler et al. [26] highlighted the importance of nutrition in analysing the welfare effects of modern technology. Tijjani et al. [42] employed various indicators to comprehensively measure the economy and well-being of female farmers in Nigeria. These included entrepreneurial opportunity, isolation, linkages to inputs and markets, aiding small- and medium-sized businesses, alleviating poverty, skill, farmers’ ability to make economic decisions, and the rate of illiteracy. A contemporary study [29] additionally adopted social development, progress, and improvement of the ecological environment as indicators for measuring social and ecological well-being.

Lastly, environmental well-being is categorized into three main subthemes: environmental quality, agricultural practices, and sustainable practices. Environmental quality is a generic term that refers to the quality of the environment, whereas environmental externalities are described as the unintentional effects of economic activities on non-market participants. Specifically, environmental externalities are the costs or benefits of economic activity that are not considered by the people or businesses in charge and are not reflected in market prices. Fiscal tools can be used to support a strong industrial innovation activity where the negative externalities of innovation on the common environment are reduced, and the impact of innovation activities on productivity is increased [61]. Hadush [16] analyses specific environmental well-being indicators in rural Ethiopia. The author measures the effect of technology adoption on farmers’ economic, ecological and social well-being using an endogenous regression model. Specifically, the author measured the hired labour, propensity to purchase extra animal feed and total cattle stock. This further included number of plants purchased, propensity to grow trees, probability of keeping milking cows, and risk of animal death.

## 4 Discussion

Table 8 summarizes the significant findings on the influence of technology adoption on farmers' well-being present throughout the SLR process, and the future research directions. This section further discusses the possible aspects of future research on the influence of technology adoption on farmers’ well-being in terms of the well-being concept and type of research.

**Table 8:** Summary table of conclusions and future research.

Theme	Main conclusion	Future lines of research
Sustainable Agricultural Practices and Innovations	● Sustainable agricultural practices and innovations were measured using various mechanisms. ● Sustainable Agricultural Practices and Innovations have shown mixed effects on the well-being of farmers. ● There are no specific well-being indicators measured by past studies to measure overall welfare. ● Most studies examined a single-country sample in developing countries.	● Provide a comparison with developed countries. ● Test specific sustainable agricultural practices and innovations on farmers' well-being.
Digital Agriculture and Information Technology	● Digital agriculture and information technology that incorporate modern technology include digital farming, digital technology, decision trees, and SVM and ICT. ● Most studies used income and productivity to measure the well-being of farmers. Past studies have not measured any specific well-being indicator of overall welfare. ● Most studies used quantitative methods. ● The data used were from single-country samples and mostly from developing countries.	● Compare developed and developing countries such as Europe, MENA, South Africa, and other Asian countries. ● Analyse data using mixed methods or qualitative studies. ● Include another aspect of well-being rather than only the net income and productivity of farmers.
Precision Farming and Resource Management	● There are five technology mechanisms under this theme: shale development, IIAD, bio fertilizer, cassava and maize technology, and mechanical tomato harvesting. ● Positive results on the effect of Precision Farming and Resource Management on farmers' well-being. ● Most studies that measured Precision Farming and Resource Management were conducted in single-country samples in the US and African regions.	● Analyse other countries such as Asian and European countries. ● Test specific mechanisms to measure farmers' well-being. ● Analyse more multi-country samples. ● Employ mixed-method studies.
Financial Inclusion and Agricultural Transactions	● Mixed results on the effect of mobile money on farmers' well-being. ● The study was conducted in Rwanda and Kenya. ● Used qualitative technique.	● Analyse multi-country samples. ● Employ mixed-method studies.
Remote Sensing and Satellite Technology	● The study resulted in not significant effect. ● Past studies only consider studying in the Czech Republic.	● Measure comprehensively on the effect of remote sensing and satellite technology on well-being by employing a mixed-method study.
Agricultural Programs and Initiatives	● Mixed results on the effect of agricultural programs and initiatives on farmers' well-being. ● Past studies only considered the WAAP Program and agricultural innovation.	● Analyse data using mixed methods or qualitative studies. ● Test specific mechanisms to measure farmers' well-being.

### 4.1 Well-being concept

Most studies have used income and productivity to measure farmers’ well-being. Mossie et al. [55] however suggested that household consumption is also a good indicator since consumption expenditure provides more reliable results than income and productivity, and is less prone to measurement errors [55]. In addition, well-being is beyond the economic aspect of income, productivity, output, or consumption since it includes value judgments such as happiness, and thus cannot be statistically measured. Therefore, if a study does not use the best indicator to measure well-being, the result may be erroneous.

Most of past studies systematically reviewed have neglected the value judgment of farmers and thus excluded important criteria of well-being, such as consumption. The definition and measurement of farmers’ well-being are essential for policymakers and practitioners in creating the most suitable policy and program to support the agricultural sector and reduce anticipated negative impact of an economic downturn [56]. Therefore, we propose that future research should conceptualize the definition and criteria of well-being to explain it more precisely and concisely.

### 4.2 Type of research

Most of the studies reviewed (21/30) relied on empirical evidence to demonstrate the connection between technology and farmers' well-being. Only seven papers employed qualitative research methods and two employed mixed methods. Qualitative research involves various authentic sources, including case studies, personal experiences, introspective interviews, and observational, historical, and visual texts that describe complex events and meanings in individuals' lives. It also involves determining what people think and how they feel. The qualitative studies thus evaluate the complex relationship between technology and farmers' well-being. Moreover, such studies could help gain better information on the overall welfare of farmers. Given the scarcity of qualitative studies examining the influence of technology adoption on farmers’ well-being, we urge future research to employ this approach.

## 5 Conclusion

The primary objective of this SLR is to examine the influence of technology adoption on farmers' well-being. In particular, we focus on these concepts by systematically reviewing previous studies on the subject. We thus classified technology into sustainable agricultural practices and innovations, digital agriculture and information technology, precision farming and resource management, financial inclusion and agricultural transactions, remote sensing and satellite technology, and agricultural programs and initiatives. Although we observed mixed results, most studies indicated that technology adoption improved farmers' well-being as measured by productivity and income, although these measures excluded value judgments by farmers, such as happiness. However, several studies have used other concepts of well-being, including per-capita consumption, environmental quality, and consumer/producer surplus, which are important criteria for well-being. Agricultural technology may have a mixed effect on farmers' well-being, depending on the type of technology adopted and the compatibility of farmers with technology in their agricultural practices. Most studies were conducted on a single country while cross-country studies focused primarily on developing countries. Many studies have applied quantitative approaches while very few adopted the qualitative method. Opportunities for future research should include aspects of well-being other than productivity or net income, examine specific technology on farmers’ well-being, conduct comparative studies between developing and developed countries, and adopt a mixed-method approach that includes both quantitative and qualitative techniques. A qualitative approach could provide more detailed information on the overall welfare of farmers.

We intentionally employed a systematic approach by considering all feasible procedures required to perform a good and reliable SLR. Therefore, we argue that the risk to validity and dependability can be kept below acceptable limits. We however limited our study to those reported in journals indexed in Scopus and WoS. Findings from other indexes should also be included in the future.

## Data availability statement

Data will be made available on request.

## Ethical declaration

Review and/or approval by an ethics committee was not required for this study because it involved a comprehensive review and analysis of existing literature, without any direct involvement with human subjects, animals, or sensitive data.

## CRediT authorship contribution statement

**Mariani Abdul-Majid:** Writing – review & editing, Validation, Supervision, Resources, Project administration, Methodology, Investigation, Funding acquisition, Formal analysis, Conceptualization. **Siti Aisyah Zahari:** Writing – original draft, Visualization, Software, Methodology, Investigation, Formal analysis, Data curation. **Norfaizah Othman:** Writing – review & editing, Validation, Supervision, Resources, Project administration, Investigation, Funding acquisition. **Suhaila Nadzri:** Writing – review & editing, Validation, Resources, Investigation, Conceptualization.

## Declaration of competing interest

The authors declare that they have no known competing financial interests or personal relationships that could have appeared to influence the work reported in this paper.

## Figures and Tables

**Table:** List of reviewed papers.

No.	Publication Year	Author	Title
1.	2022	Bambio Y., Deb A., Kazianga H.	Exposure to agricultural technologies and their adoption: The West Africa agricultural productivity program in Ghana, Senegal, and Mali
2.	2022	Baiyegunhi L.J.S., Akinbosoye F., Bello L.O.	Welfare impact of improved maize varieties adoption and crop diversification practices among smallholder maize farmers in Ogun State, Nigeria
3.	2021	Liang, Zhihui; Zhang, Lu; Li, Wenjing; Zhang, Junbiao; Frewer, Lynn J.	Adoption of combinations of adaptive and mitigatory climate-smart agricultural practices and its impacts on rice yield and income: Empirical evidence from Hubei, China
4.	2021	Yang D., Zhang H.-W., Liu Z.-M., Zeng Q.	Do cooperatives participation and technology adoption improve farmers' welfare in China? A joint analysis accounting for selection bias
5.	2021	Akkaya D., Bimpikis K., Lee H.	Government interventions to promote agricultural innovation
6.	2021	Uwamariya M., Loebbecke C., Cremer S.	Mobile money adoption in rural Rwanda: A domestication perspective
7.	2021	Carter M., Laajaj R., Yang D.	Subsidies and the African Green Revolution: Direct Effects and Social Network Spillovers of Randomized Input Subsidies in Mozambique†
8.	2021	Hadush M.	Does it pay to switch from free grazing to stall feeding? Impact of stall feeding practice on household welfare in Tigrai Ethiopia
9.	2020	Yang X., Sang Y.	How does part-time farming affect farmers' adoption of conservation agriculture in jianghan plain, china?
10.	2020	Řezník T., Pavelka T., Herman L., Lukas V., Širůcěk P., Leitgeb S., Leitner F.	Prediction of yield productivity zones from Landsat 8 and Sentinel-2A/B and their evaluation using farm machinery measurements
11.	2020	Marshall A., Dezuanni M., Burgess J., Thomas J., Wilson C.K.	Australian farmers left behind in the digital economy – Insights from the Australian Digital Inclusion Index
12.	2020	Wulandari D., Utomo S.H., Narmaditya B.S., Priambodo M.P., Prayitno P.H.	Digital farming and local economy: A study on nudira, greenhouse bandung
13.	2020	Goeb J., Dillon A., Lupi F., Tschirley D.	Pesticides: What you don't know can hurt you
14.	2020	Sowmya B.J., Krishna Chaitanya S., Seema S., Srinivasa K.G.	Data analytic techniques for developing decision support system on agrometeorological parameters for farmers
15.	2019	Crowe J.A.	The impact of shale development on crop farmers: how the size and location of farms matter
16.	2019	Keil A., Mitra A., Srivastava A.K., Mcdonald A.	Social inclusion increases with time for zero-tillage wheat in the Eastern Indo-Gangetic Plains
17.	2018	Dyck, B; Silvestre, Bs	Enhancing socio-ecological value creation through sustainable innovation 2.0: Moving away from maximizing financial value capture
18.	2017	Tijjani A.R., Anaeto F.C., Emerhirhi E.	Analysis of the roles of information and communications technologies in rural women farmers' empowerment in Rivers State, Nigeria
19.	2017	Athukorala W., Wilson C.	Distributional impacts of irrigation-induced agricultural development in a semi-subsistence economy: new evidence
20.	2017	Afrin S., Haider M.Z., Islam M.S.	Optimal use of pesticide for paddy production in the south-west region of Bangladesh
21.	2017	Wesseler J., Smart R.D., Thomson J., Zilberman D.	Foregone benefits of important food crop improvements in Sub-Saharan Africa
22.	2016	Barragán-Ocaña A., Del-Valle-Rivera M.C.	Rural development and environmental protection through the use of biofertilizers in agriculture: An alternative for underdeveloped countries?
23.	2015	Smyth S., Kerr W., Phillips P.W.B.	The unintended consequences of technological change: Winners and losers from GM technologies and the policy response in the organic food market
24.	2014	Kikulwe E.M., Fischer E., Qaim M.	Mobile money, smallholder farmers, and household welfare in Kenya
25.	2014	Smale, M; Mason, N	Hybrid Seed and the Economic Well-Being of Smallholder Maize Farmers in Zambia
26.	2005	Jackson L.A., Anderson K.	What's behind GM food trade disputes?
27.	2005	Lucia Omobolanle, O	Resourse use pattern and farmers' productivity in southwest Nigeria
28.	1998	Nelson R.A., Cramb R.A.	Economic incentives for farmers in the Philippine uplands to adopt hedgerow intercropping
29.	1995	Lighthall D.R., Roberts R.S.	Towards an alternative logic of technological change: Insights from Corn Belt agriculture
30.	1970	Schmitz A., Seckler D.	Mechanized agriculture and social welfare: The case of the tomato harvester
